# The effects of sleep loss on young drivers’ performance: A systematic review

**DOI:** 10.1371/journal.pone.0184002

**Published:** 2017-08-31

**Authors:** Shamsi Shekari Soleimanloo, Melanie J. White, Veronica Garcia-Hansen, Simon S. Smith

**Affiliations:** 1 Centre for Accident Research and Road Safety, Queensland University of Technology, Brisbane, Queensland, Australia; 2 School of Psychology and Counselling, Queensland University of Technology, Brisbane, Queensland, Australia; 3 Institute of Health and Biomedical Innovation, Queensland University of Technology, Brisbane, Queensland, Australia; 4 School of Design, Queensland University of Technology, Brisbane, Queensland, Australia; 5 Recover Injury Research Centre, UQ Oral Health Centre, The University of Queensland, Brisbane, Queensland, Australia; Beihang University, CHINA

## Abstract

Young drivers (18–24 years) are over-represented in sleep-related crashes (comprising one in five fatal crashes in developed countries) primarily due to decreased sleep opportunity, lower tolerance for sleep loss, and ongoing maturation of brain areas associated with driving-related decision making. Impaired driving performance is the proximal reason for most car crashes. There is still a limited body of evidence examining the effects of sleep loss on young drivers’ performance, with discrepancies in the methodologies used, and in the definition of outcomes. This study aimed to identify the direction and magnitude of the effects of sleep loss on young drivers’ performance, and to appraise the quality of current evidence via a systematic review. Based on the Preferred Reporting Items for Systematic Reviews and Meta- Analyses (PRISMA) approach, 16 eligible studies were selected for review, and their findings summarised. Next, critical elements of these studies were identified, and the Grading of Recommendations Assessment, Development and Evaluation (GRADE) guidelines augmented to rate those elements. Using those criteria, the quality of individual papers was calculated and the overall body of evidence for each driving outcome were assigned a quality ranking (from ‘very low’ to ‘high-quality’). Two metrics, the standard deviation of lateral position and number of line crossings, were commonly reported outcomes (although in an overall ‘low-quality’ body of evidence), with significant impairments after sleep loss identified in 50% of studies. While speed-related outcomes and crash events (also with very low- quality evidence) both increased under chronic sleep loss, discrepant findings were reported under conditions of acute total sleep deprivation. It is crucial to obtain more reliable data about the effects of sleep loss on young drivers’ performance by using higher quality experimental designs, adopting common protocols, and the use of consistent metrics and reporting of findings based on GRADE criteria and the PRISMA statement. Key words: Young drivers, sleep loss, driving performance, PRISMA, the GRADE, systematic review.

## Introduction

Sleepiness is a primary cause of road crashes [1–3], underlying an average of 20% of all crashes in developed countries [2, 4–8] (17% in Australia [9, 10], and 25% in the UK [11, 12]). Road crashes impose a huge economic and social burden, estimated to be $1,855 billion per year globally [13] on modern societies. Based on a conservative estimates derived from police reports, sleep-related crashes cost $12.5 billion monetary losses in the US annually [14]. However, these figures are likely the tip of the iceberg, with actual costs potentially $29.2 to $37.9 billion in the USA [15]. Sleepiness is mainly induced by sleep deprivation [16] due to total sleep loss, partial sleep loss, extended wake duration, and sleep fragmentation or sleep disturbances.

Young drivers (those aged 18–24 years) are generally at higher risk for road crashes than are older drivers [2, 17–19], with an estimated risk of crash between 2 to 10 fold, when compared with other age groups [5, 20]. Young drivers also comprise a greater proportion of driver fatalities. Some specific characteristics of young drivers such as late maturation of their brains’ decision-making areas [21, 22], their slower reaction times while sleepy [23, 24], and a lower tolerance for sleep loss than older adults [25], results in greater vulnerability to sleep deprivation [26, 27] and hence their over representation in sleep-related crashes [20, 26, 28].

To the best of the authors’ knowledge only three systematic reviews of effects of sleep deprivation on driving tasks have been published. The first review examined the effect of driver sleepiness (from shift work, excessive daytime sleepiness and sleep loss) on crash rates, but not on any other specific index of driver performance [29]. This review included 18 cross sectional and case-control studies with only one paper examining the effect of sleep loss on crash rate. The papers generally could not make a robust conclusion on the relationship between fatigue and crash rate due to small sample sizes, biases, and aspects of their designs, and could not identify a strong effect of sleepiness on crash rate [29]. The second review investigated the effect of sleepiness on driving performance outcomes to determine if such outcomes could reliably predict driver sleepiness on road. This review included papers with a broad inclusion of participants, cause of sleepiness (sleep loss vs fatigue from time-on-task), driver experience (professional driver vs road user), and sleep disturbance (shift worker vs non-shift worker). They found that the majority of studies had examined simple performance measures such as standard deviation of lane position in controlled experimental settings, with results reported as an average among drivers. Individual differences were largely not taken into account[30]. A recent systematic review by the National Sleep Foundation Drowsy Driving Consensus Working Group [31] considered the severity of sleep loss and involvement in motor vehicle crash for drivers over the age of 15 years. Their consensus conclusion was that drivers would be impaired by 3 to 5 hours sleep loss incurred during the preceding 24 hours.

Apart from the above-mentioned systematic reviews, about 200 original research papers have been published on the topic of the effects of sleepiness or fatigue on driving tasks. However, the effects of sleep loss on young drivers’ performance specifically remains uncertain in that, a) more than 50% of these papers did not study sleepiness from sleep loss, but instead from other sources such as time-on task fatigue or usual daytime sleepiness, or they have examined the effect of countermeasures for sleepiness (e.g. light, modafinil, caffeine, etc.),but not the effects of sleepiness itself, and b) about 40% of papers have included a broad range of drivers (professional and non-professional, young and old drivers), or examined only the prevalence of sleepiness or outcome measures other than driving performance. Fewer than 10% of the existing literature has examined the direct effects of sleep loss on driving performance of young drivers (between 18–24 years old).

Given the higher vulnerability of young drivers to sleep related crashes, and the high cost of sleepiness-related fatalities and severe injuries it is crucial to systematically review the available body of evidence. A systematic review provides the opportunity to better understand the effects of sleep deprivation on driving performance of young drivers and to inform future prevention strategies. This paper aims to systematically review all peer-reviewed original research studies, and to rate the quality of the available body of evidence on effects of sleep deprivation on young drivers’ driving performance over the last 12 years. A preliminary search into the databases revealed that applicable and relevant data about the effects of sleep loss on driving performance outcomes in young adults specifically are largely limited to the last decade. As such, a 12-year period was defined for inclusion of relevant studies. As sleep loss is a public health problem, the research team agreed that if a meta-analysis was not feasible due to data limitations, then an appropriate evaluative approach should be taken to estimate the quality of evidence (i.e. the confidence in current knowledge).

The term ‘sleepiness’ in this paper refers to the broader term ‘fatigue’ as well. It is acknowledged that ‘sleepiness’ could be more precisely distinguished from other conceptualizations of ‘fatigue’, particularly chronic fatigue [32]. However, in the current review, due to coexistence of sleepiness and fatigue after sleep loss [33] and lack of standard definitions for these terms, the two terms have been considered interchangeably to address a ‘need for sleep’.

## Materials and methods

This systematic review was conducted by the authors based on the PRISMA statement; Preferred Reporting Items for Systematic Reviews and Meta- Analyses [34]. A protocol was developed for this systematic review, but was not registered. In the first step, following the PRISMA statement, the research question, the scope of the study and inclusion/ exclusion criteria were defined. Next, the available literature was systematically screened before selection of eligible studies based on PRISMA flowchart. Finally, the selected papers were reviewed, the quality of the body of evidence was rated and the effect sizes of sleepiness on drivers’ performance were summarised using the GRADE guidelines; Grading of Recommendations Assessment, Development and Evaluation [35–48]. Two review groups (group 1: SH.SH.S + S.S.S and group 2: M.J.W + V.G.H) conducted the review steps independently and reached a consensus before moving to the next step.

### Research question

The elements of Population, Intervention, Comparator (control), Outcomes and Study design (PICOS, [34] were considered from the PRISMA statement in development of the research question as

“What are the effects of sleep loss on young drivers’ driving performance outcome measures?”

### Scope of the review, inclusion/exclusion criteria

To answer the research question, specific inclusion and exclusion criteria were set to define inclusion of original research papers studying the independent effects of sleep deprivation on young adults’ driving performance. These criteria were based on characteristics of the papers such as peer-review status, participants, sleepiness exposure, outcome measures, publication date, and study design as well as publication language (Table 1). Because of the increased risk of bias from translation of information from other languages to English [49], and the likely modest impact of removing non-English literature on the estimation of effects [50], papers published in other languages were excluded.

**Table 1:** Inclusion criteria and exclusion conditions for selecting papers for systematic review.

Study element	Inclusion criteria	Exclusion condition
**Peer-review**	Original research papers or systematic reviews published in peer-reviewed journals	Non-peer reviewed papers, book chapters, reports, conference proceedings were excluded
**Subjects**	Participants should be young (16–26 yrs. old inclusive), healthy, non-professional driver, non-shift worker, free from sleep disorders	Papers with broader age range were excluded
**Sleepiness**	Sleepiness was induced by sleep deprivation only. Sleepiness could be induced by any type of sleep deprivation including acute or chronic sleep loss, extended wake periods, early morning wakeups (sleep limitation), sleep fragmentations or sleep disturbances	Studies examining other forms of sleepiness without any prior sleep loss (e.g. time-on task fatigue or usual daytime sleepiness) were excluded
**Exposure (independent variable)**	Sleep deprivation was the main exposure (independent variable)	Studies examining the effect of countermeasures for sleepiness (e.g. light, modafinil, caffeine, exercise, nap, alcohol, etc.) on sleep deprived subjects were excluded
**Outcome measures (dependent variable)**	The primary outcome measures of interest should include driving performance outcomes, either driving simulator or on–road. Driving performance outcomes could be studied individually or along with other objective and subjective determinants of sleepiness	
**Publication date**	Published between 1 January 2004 and 30 December 2016	
**Study design**	Any type of study design; all study designs such as Randomised Control Trials (RCTs), experiments, cross-sectional and observational studies were included	
**Publication language**	Papers published in English only	Papers published in other languages were excluded

### Search strategy and selection of studies

A comprehensive Boolean/Phrase search was conducted from the 3^rd^ to the 10^th^ of January 2017 within the electronic databases including PsycINFO (via EBSCOhost), PsycARTICLES (via EBSCOhost), MEDLINE (via EBSCOhost), Science Direct, ProQuest Psychology, Web of Science, Scopus, Ergonomic Abstracts (via EBSCOhost), PubMed (via NCBI), Trip (Turning Research into Practice), CINAHAL (via EBSCOhost), Transportation Research Information Database, The Cochrane Library, EMBASE and Academic Search Elite (via EBSCOhost).

A specific search statement was developed as follows: [(“sleep depriv*” OR “sleep loss” OR “sleep limitation” Or “sleep restriction”) AND (“sleepiness” OR drows* OR hypersomnol* OR “sleep onset” OR “excessive sleep*” OR “sleep propensity” OR fatigue* OR microsleep* OR alert* OR vigilance OR hypovigilan*) AND (driver OR simulator OR vehicle OR “commercial driver” OR “professional driver” OR “driver performance” OR “truck driver” OR “bus driver”)].

Some databases, such as Transportation Research Information Database, The Cochrane Library and EMBASE, do not utilise asterisk (*) within their search strategy. AS such, in the search statement the complete wordings of key words were utilised for these databases. By using some filters, the records were narrowed to include only peer-reviewed papers published within the last 12 years (from 2004 to 2016). In some cases, the journal websites were checked directly to ensure peer-review processes. The search (via the above databases) was restricted further to English language only. Search alerts were activated where available to automatically update the records. Bibliographic records of all identified papers were also examined to identify additional potential papers for inclusion.

Using the PRISMA 2009 flow diagram [34], all potential papers were first identified via this search strategy. After aggregating all records and removing duplicates, screening of the title and abstracts of all papers against inclusion criteria was undertaken by two review groups independently. The full-text prints of selected papers were assessed for eligibility and the reason for inclusion/exclusion of papers was recorded by the review groups independently. Finally, papers were selected by a discussion with other members of the research team, and a consensus approach was used to decide in case of any discrepancy. Where required, further information was sought from authors of selected papers about their research to inform these decisions.

### Summarising the papers

Based on the GRADE guidelines [37, 39, 40], the important elements of selected studies were summarised and criteria for rating the quality of the papers were developed. For this purpose, some specific and important aspects of individual papers such as study design/objective, sample size, participants’ age range, sleep deprivation regime, driving settings, driving duration, frequency and time of drive, and driving performance outcomes were reviewed and the important methodological elements (strengths and potential flaws of the studies) were extracted and summarised. Not all items specified in the GRADE (a schema developed primarily for review of health and medical literature) are applicable to studies on road safety, as such, adaptation was needed to apply GRADE to this literature (i.e. experimental studies versus RCTs etc.). Also based on the possible differential consequences of various degrees of sleep deprivation [51–53], the sleep deprivation regimens were classified into acute and chronic sleep loss, with acute sleep loss rated at three levels of moderate (2–4 h), severe (4–6 h) and total (8 h) sleep loss, and chronic sleep loss rated at two levels of mild (1–2 h) and moderate (2–4 h) sleep loss.

### Development of the GRADE criteria

The GRADE guidelines [37, 39, 40] include some criteria for rating the quality of the papers. GRADE is a flexible approach and relies to some extent upon the judgment of the researcher, as such, additional criteria were derived from the summarised aspects of the studies and their methodological elements in order to augment the existing GRADE criteria. These modified GRADE criteria were comprised of discipline-specific downgrading and upgrading scores for rating the quality of the reviewed papers.

### Identification of the quality the body of evidence

Using the modified GRADE criteria and the GRADE guidelines [37], a multi-step approach was taken to identify the quality body of evidence for the outcomes: First, these modified GRADE criteria were utilised to calculate a single GRADE score for every outcome measure reported in each individual papers. Next, these single GRADE scores were utilised to calculate an overall quality of evidence for all papers reporting the same outcome. Finally, a quality rank was assigned to the body of evidence for every driving performance outcome.

#### Rating the quality of individual papers

The quality of a driving performance outcome measure was rated in individual papers by considering factors degrading the quality of papers including poor study design, risk of bias (due to inadequate monitoring sleepiness during test (wake EEG) and presence of practice effect), and imprecision (due to ungeneralizable findings and small sample size), as well as some upgrading factors including large effect size, large sample size, objective measurement of sleepiness (EEG) and control for distraction. For this purpose, a four-step approach was taken as follows: 1) as for the study design, the GRADE score of 4, 2, 1 and 0 were first assigned to studies with randomised control trial (RCTs), longitudinal, quasi experimental, and other designs respectively. In the sleep studies, quasi-experimental designs that manipulate sleep and longitudinal studies that provide detail of the cumulative effects of chronic sleep deprivation are both capable of showing the magnitude and direction of effect of sleep loss on drivers’ performance. Therefore, the GRADE scores were modified by adding one point to studies applying either of these two designs. 2) The quality of the papers was further assessed for risk of bias and imprecision. Given that the risk of bias and imprecision adversely affect measurement of driving performance outcomes and the generalizability of the findings, the quality of the papers was downgraded by deducting one point for existing risk of bias (e.g. inadequate monitoring of sleepiness during driving task, presence of practice effect), and by further deduction of one point for imprecision (e.g. increased uncertainty due to small sample size). 3) The quality of papers was upgraded by adding one point for their methodological strengths such as strong control of sleep loss before test and by an additional point for factors increasing certainty of findings. 4) A single quality score was assigned to the individual papers by adding all positive and negative points in the above-mentioned order. The same process was repeated for other outcome measures of driving performance.

#### Rating the quality of body of evidence

Based on the single GRADE scores of individual papers, an Overall GRADE Score (OGS) was calculated for the body of evidence (including at least two individual papers reporting the same outcome). It should be noted that the OGS for the body of evidence was not determined by averaging the single GRADE scores, but by considering the contribution of individual papers toward the estimated magnitude of effect of sleep loss on a given driving performance outcome. For example, studies with larger sample sizes were considered as more important contributors, and were weighted to reflect that contribution. There is no recommended algorithm in the GRADE guidelines to calculate the OGS for the body of evidence. As such, a new formula including the sample size was developed to calculate the OGS as follows:

$$\text{Overall Grade Score for the body of evidence }=\;\frac{\sum \left(\text{GRADE score for paper * Sample size of paper}\right)}{\text{Total sample size of the body of evidence}}$$

#### Ranking the quality of body of evidence

The quality of body of evidence for each outcome was ranked, by review team consensus, at four ranking levels from ‘very low’ to ‘high-quality’ based on the GRADE guidelines [37].These ranks reflect the extent of confidence that the estimated effect is close to the true effect. The GRADE guidelines [37] do not directly map onto the OGS for the body of evidence at the above-mentioned levels, so four ranges of OSG scores were assigned to these four quality rank (based on judgment of the research team) as follows:

1. High quality (3≤ OGS): a high confidence of true effect lying close to the estimated effect,
2. Medium quality (2≤ OGS <3): a moderate confidence of true effect lying close to the estimated effect,
3. Low quality (1≤ OGS <2): a limited confidence of true effect lying close to the estimated effect,
4. Very low quality (0≤ OGS <1): a very little confidence of true effect lying close to the estimated effect.

Using these grading and ranking protocols the two review groups first graded and ranked the studies independently before a group discussion to ensure consensus.

## Results

### Database search and data extraction

**Fig 1:**
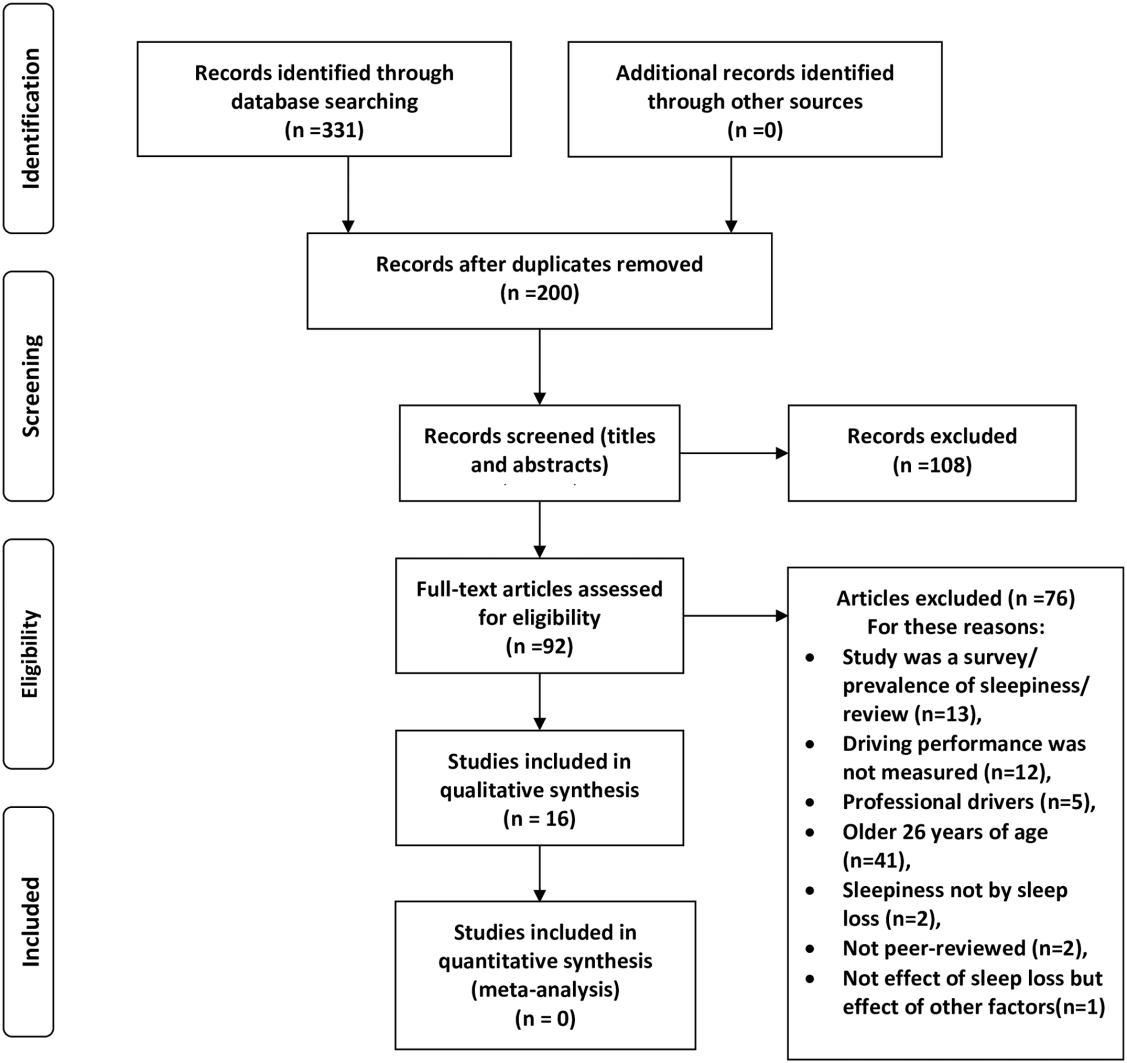
Flow diagram of systematic review based on PRISMA 2009.

Table 2 shows the search statement and number of papers initially selected from individual databases. Initially, 331 records were identified through an online search into the 15 electronic databases. From these 331 papers, 131 duplicate papers were removed. The titles and abstracts of 200 remaining papers were screened, and 108 irrelevant records were excluded. The majority of these 200 papers (more than 50%) did not address the implications of sleep loss on adults’ performance, instead they studied effects of time-on task fatigue, usual daytime sleepiness or Obstructive Sleep Apnoea on drivers’ performance, or they have examined the effects of nap, light, wake-promoting agents, caffeine, etc. on drivers’ sleepiness. The full texts of the 92 remaining records were assessed and 76 papers (more than 40% of the primary 200 papers) were excluded as they studied professional drivers, or the prevalence of sleepy driving only, or did not include driving performance outcomes in their designs. Finally, the 16 remaining papers (only 8% of the primary selected papers) were included in the systematic review. It should be noted that despite the presence of some other sleepiness-related studies that included the same age group [54, 55], these studies could not be included since the outcome measures did not include driving performance [54], or their sample included older adults as well [55]. Fig 1 presents the data extraction flowchart including the reasons for excluding papers.

**Table 2:** Search statements and limiters and number of papers identified from each database.

No	Database	Search Dates	Search Statement/limiters	Search identified records	Primary selected records
**1**	Transportation Research Information Documentation (TRID)	3/1/2017	Statement (1)^a^, Limiters: Publication type: Publications; Language: English; Publication date: 200401 to 201612	159	63
**2**	PsycINFO (via EBSCOhost)	4/1/2017	Statement (2)^b^, Limiters: Peer Reviewed; Published Date: 20040101–20161231; Language: English; Age Groups: Young Adulthood (18–29 yrs.); Population Group: Human; Search modes—Boolean/Phrase	60	15
**3**	PsycARTICLES (via EBSCOhost)	4/1/2017	Statement (2), Limiters—Year of Publication: 2004–2016; Published Date: 20040101–20161231; Scholarly (Peer Reviewed) Journals; Age Groups: Young Adulthood (18–29 yrs.); Population Group: Human; Expanders—Also search within the full text of the articles	37	0
**4**	MEDLINE (via EBSCOhost)	4/1/2017	Statement (2), Limiters: Date of Publication: 20040101–20161231; English Language; Narrow by subject age: adult: 19–44 yrs. Search modes—Boolean/Phrase	120	21
**5**	ScienceDirect	5/1/2017	Statement (2) Limiters: Only Journals; all sources; all sciences; From 2004 to present	78	1
**6**	ProQuest Psychology	5/1/2017	Statement (2) Limited by: Date: From 01 January 2004 to 31 December 2016 Source type: Books, Dissertations & Theses, Scholarly Journals Document type:11 types searched Article, Book, Book Chapter, Case Study, Conference, Conference Paper, Conference Proceeding, Evidence Based Healthcare, Literature Review, Review, Technical Report Language: English	38	1
**7**	Web of Science	6/1/2017	Statement (2) Language: (English); Document Types: Article; Timespan: 2004–2017. Indexes: SCI-EXPANDED, SSCI, CPCI-S, CPCI-SSH, BKCI-S, BKCI-SSH.	295	27
**8**	Scopus	8/1/2017	Statement (2) Exclude key words: Middle Aged, Sleep Disorder, Work Schedule Tolerance, Work Schedule Limit subject area: Medicine, Neuroscience, Social Sciences, Psychology, Engineering, Health Professions, Computer Science, Environmental Science, Multidisciplinary, Decision Sciences, Limit Document Type: Article, Article in press, Erratum Limit Language: English Publication year 2004 to present	1781	91
**9**	Ergonomic Abstracts (via EBSCOhost	5/1/2017	Statement (2) using smart search Limiters—Scholarly (Peer Reviewed) Journals; Publication Date: 20040101–20161231 Search modes—Boolean/Phrase	36	14
**10**	PubMed in NCBI	7/1/2017	Statement (2) Additional filters: publication date from 1/01/2004 to 31/12/2016, Language: English	161	22
**11**	The Cochrane Library	8/1/2017	Statement (1) Publication Year from 2004 to 2016; Word variations have been searched	26	3
**12**	TRIP (Turning Research into Practice)	10/1/2017	Statement (1) From:2004 to:2016	210	8
**13**	EMBASE	8/1/2017	Statement (1) Publication date from 2004 to 2016	263	34
**14**	CINAHAL (via EBSCOhost)	5/1/2017	Statement (2) Limiters—Published Date: 20040101–20161231; English Language; Peer Reviewed; Human; Age Groups: Adolescent: 13–18 yrs., Adult: 19–44 years; Language: English Search modes—Boolean/Phrase	10	0
**15**	Academic Search Elite (via EBSCOhost)	4/1/2017	Statement (2) Limiters—Published Date: 20040101–20161231; Scholarly (Peer Reviewed) Journals; Language: English Expanders—Also search within the full text of the articles Search modes—Boolean/Phrase	692	31
**Total**				3935	331

^a^ Statement (1): (driver or simulator or vehicle or "commercial driver" or "Professional driver" or "driver performance" or "truck driver" or "bus driver") and (sleepiness or drowsiness or hypersomnolence or "sleep onset" or "excessive sleepiness" or "sleep propensity" or fatigue or microsleep or alertness or vigilance or hypovigilance) and ("sleep deprivation" or "sleep loss" or "sleep limitation" or "sleep restriction"),

^b^ Statement (2): (“sleep depriv*” OR “sleep loss” OR “sleep limitation” Or “sleep restriction”) AND TX ((“sleepiness” OR drows* OR hypersomnol* OR “sleep onset” OR “excessive sleep*” OR “sleep propensity” OR fatigue* OR microsleep* OR alert* OR vigilance OR hypovigilan*) AND TX (driver OR simulator OR vehicle OR “commercial drivers” OR “professional driver” OR “driver performance” OR “truck driver” OR “bus driver”)

### Designs and methodologies

**Fig 2:**
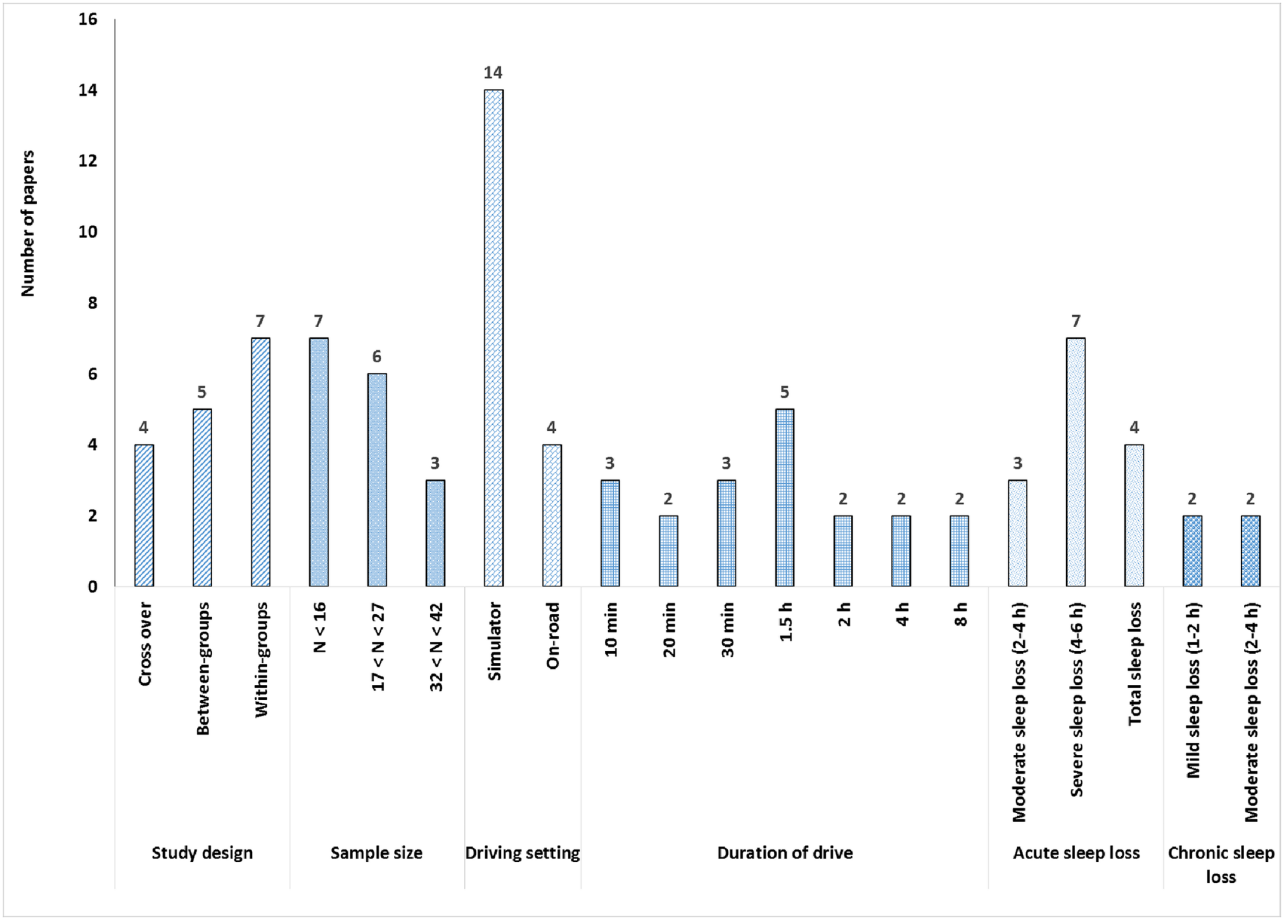
Distribution of papers based on their methodological elements.

Fig 2 presents the distribution of reviewed papers based on their methodological profiles. There were no randomised control trials within the reviewed papers. There was a homogenous group of experimental studies including 4 cross-over studies [56–59], 5 between-groups [26, 60–63], and 7 within-group [64–70] designs.

The sample sizes ranged from 8 to 41 participants, with 7 papers having samples of fewer than 16 participants [56, 58, 59, 64, 66, 68, 70], 6 papers with 17–27 participants [26, 57, 61, 65, 67, 69], and 3 papers having a sample size greater between 32 and 42 [60, 62, 63]. Male participants were overrepresented in all samples, with half of the studies (8 papers) only recruiting males.

Of the 16 studies, only 4 studies were conducted on real roadways ([56–59], with two of those studies also including simulated drives in their protocol [56, 57]. The remaining 12 studies utilised a driving simulator only.

Driving durations ranged from 10 minutes to 8 hours. Overall, 50% of studies (8 papers) adopted short durations of less than 30 minutes (10 minutes: [60, 63, 66], 20 minutes; [67, 69], and 30 minutes [61, 65, 68, 70]. The other 8 papers varied in the durations of their drives, with some of studies examining multiple drive durations in their protocols. Only two papers, reporting data from the same study, adopted longer driving durations of 4 and 8 hours [58, 59].

The majority of the reviewed studies (12 papers) adopted an acute sleep loss protocol, with 3 papers exerting a moderate (between 2 and 4 hours) sleep loss [61, 62, 64], 7 papers severe sleep loss [61] [26, 56–59, 64], and 4 papers exerting total sleep loss [65, 68–70]. The remaining 4 papers included a chronic sleep deprivation paradigm [60, 63, 66, 67].

Table 3 presents a detailed summary of key methodological characteristics of individual papers including year and country of publication, design and objectives, sample size, participant age, sleep deprivation regime, driving setting and driving duration, frequency and time of day when driving, as well as driving performance outcome measures.

**Table 3:** Summary of key methodological characteristics and findings of individual papers.

Paper Author(s)	Country/Year	Design/objective	Sample /gender	Age range	Sleep loss	Drive setting/ drive duration	Frequency /time (s) of drive	Driving performance outcomes (specific definition)	Findings/effect size
**Acute sleep loss**									
Moderate (2 h <sleep loss <4h) and severe (4h <sleep loss <6h)									
Rupp, et al., 2004)	US /2004	Repeated measures between- groups design, comparison of normal sleep with moderate and severe sleep loss (5 and 3 h sleep respectively) while doing single driving or dual driving and subtraction tasks.	26 (13 men and 13 women)	18–26 yrs.	Sleep loss for 3 h, and Sleep for 5 h	Simulator/30 min	Night time from 1a.m and 9 a.m., and from 3 a.m. and 9 a.m.	a) Lateral position, lane deviation (deviation in road position from lane centre), b) SD ^a^ of lateral position (lane variability), c) Lane crossings (car crosses one of lane markers), d) Lane incidents (count of times car crossed into lane edge), e) mean deviation from speed limit, f) SD of deviation from speed limit (Speed variability), g) Wind reaction time: seconds taken to correct road position in presence of wind gust	a) No significant main effects or interactions on lane deviation (F = 0.3), b) Interaction of group and task type: greater lane variability for sleep deprived group for the dual driving and subtraction task versus the single driving task (medium effect size, Cohen’s d = 0.79). c) Interaction of group and session: greater lane variability for sleep deprived group vs the control group, Cohen’s d = 0.85 (large effect size), e) No effect on mean and SD of deviation from speed limit (F = 0.58, P > 0.05) and (F = 3.47, P > 0.05), f) Increase of 1.4 in lane crossings after sleep loss for single driving task, g) Increase of 1.6 in lane crossings after sleep loss on dual driving and subtracting task, h) Interaction of sleep condition and experimental session on lane crossings (large effect size; Cohen’s d = 0.98).
(Philip, et al., 2005_(b)_)	France /2004	Quasi-experimental (Cross-over), comparison of 6 times of drives on real road and simulator after habitual sleeps (8 h) with those after only sleep for 2 h in 6 times of the day.	12 men	19–24 yrs.	Sleep for 2 h, from 11p.m. to 1 a.m.)	a) On a highway b) on a Divided Attention Steering Simulator/ 1.5 h	6 times/ day between 9 a.m. to 9:30 p.m.	a) Lateral position (car distance from lateral lanes), b) Line crossing (car crosses one of lane markers).	a) Lateral position not reported, b) Main effect of sleep loss (8- fold increase) on inappropriate line crossings (F_1,10_ = 60.013, P < .001) On the simulator, c) No effect of time of day on lane crossing (F_5,50_ = 1.274, P = 0.301) on the simulator, d) On real road, an increase of 8 cases in line crossing compared with no line crossing in rested condition.
(Philip, et al., 2005(a))	France /2005	Randomized open cross-over design, comparison of 5 times of real driving after habitual sleep (8 h) with those after only sleep for 2 hours	22 men	18–24 yrs.	Sleep for 2 hours, from 11 p.m. to 1 a.m.)	Open Highway/1.5 h	5 times/ day from 9 a.m. to 7:30 p.m.	a) Lateral position (car distance from lateral lanes), b) Lane deviation (mean lateral deviation from centre of the road), c) Line crossing (car crosses one of lane markers).	a) Lateral position not reported, b) Lane deviation not reported, c) Cumulative number of line crossing after 5 times of drives Increased from 66 cases (rested) to 535 cases (sleep-restricted); (incidence rate ratio (IRR): 8.1(95% CI): 3.2–20.5; p < 0.001).
(Lowden, et al., 2009)	Sweden /2008	Repeated measures Between-subjects design, Comparison of performance of young and elderly drivers after extended wake time.	10 young (5 male, 5 female), 10 elderlies (5 male, 5 female).	18–24 yrs., 55–64 yrs.	5.5 h (extended wake) and 2 h sleep.	Hi-Fi moving base simulator/1.5 h	Single drive either in afternoon or at night time.	a) Lateral position (perpendicular distance between the right side of the right front wheel and the left side of the right-hand lane boarder), b) SD of lateral position, c) Mean and SD of speed,	a) Lateral position not reported, b) Mean and SD of speed not reported, c) SD of lateral position increased from the 30^th^ minutes of drive onwards.
(Sagaspe^b^, et al., 2008), (Verster, et al., 2011)	France /2008	Quasi-experimental (Cross-over), comparison effects of 2, 4 and 8 h sleep loss (extended wake)	14 men	21–25 yrs.	2, 4 and 8 h sleep loss (extended wake)	Two- lane highway/2 h, 4 h, 8 h	Reference session (9–10 p.m.), Midnight at the wake time	Inappropriate line crossing, lane crossing (car crosses one of lateral lane markers)	The incidence rate ratios of inappropriate line crossings, compared to the reference session (9–10 p.m.), were 6.0 (95% CI, 2.3 to 15.5; P,.001), 15.4 (CI, 4.6 to 51.5; P,.001) and 24.3 (CI, 7.4 to 79.5; P,.001), for 2 h, 4h and 8 h driving durations respectively.
(Filtness, et al., 2012)	UK /2011	Repeated measures between- subjects, comparing of sleep in normal and restricted to 5 h (extended wake) conditions among young and elderly drivers	20 young men, 20 old men	20–26 yrs., 52–74 yrs.	Sleep loss (extended wake) for 3 h	Immobile car with a computer- generated road projection/1.5 h	afternoon	Lane crossings; lane departure (all four wheels came out of the driving lane)	Interaction of sleep condition and age group; lane crossing increased in the last 30 min of the drive in both young and old drivers, with more impairment in young drivers.
(Anderson & Horne, 2013)	UK /2012	Repeated measures within-subjects design, comparison of driving after normal sleep and extended wake	8 men	20–26 yrs.	Sleep loss (extended wake) for 3 h,	Immobile car with a computer-generated road projection/2 h	Afternoon at 2 p.m.	a) Driving incidents; lane crossings (when at least two wheels of the vehicle leaving the carriageway), b) Distraction	a) positive correlation between number of distractions and number of lane crossings under sleep restriction (large effect size r = 0.74); b) from 2308 distractions under sleep deprivation 474 distraction directly resulted in incidents (t = 2.73; df = 7; p < 0.03)
Total sleep loss (6 <sleep loss <8)									
(Pizza, et al^c^., 2004),	Italy /2004	Repeated measures within-subjects design, comparison of normal sleep and sleep deprivation.	10 (5 men and 5 women)	Mean 24.9 (± 0.6) yrs.	One night total sleep loss	STISIM 300 Driving Simulator/30 min	4 times/day between morning and afternoon	a) Lateral position (distance from the car to the left lane marker), b) Mean lane position (mean distance from lane centre), c) Lane position variability; SD of lateral position (deviation in distance from the car to the midline), d) Number of crashes, e) Mean and SD of speed, f) Deviation from the speed limit.	a) Lateral position not reported, b) No change in mean lane position across drives (χ2 = 0.99), c) Increase of lane position variability with the highest of 0.20 at 2 p.m. (χ2 = 0.003, p<0.05), d) Increased of number of crashes with the highest of 0.8 at 2 p.m., e) No change in mean and SD of speed, even worsening of mean speed (χ2 = 0.98, p > 0.05) and the SD of speed (χ2 = 0.21, p > 0.05).
(Contardi, et al., 2004)	Italy /2004	Repeated measures within-subjects design, Comparison normal sleep and sleep deprivation.	10 (5 men and 5 women)	Mean 24.9 (± 0.6) yrs.	One night total sleep loss	STISIM 300 Driving Simulator/30 min	4 times/day between morning and afternoon	a) Lateral position, b) SD of lateral position, c) Number of crashes, d) mean and SD of speed, e) Deviation from the speed limit (frequency of exceeding the speed limit 120km/h).	Increase in deviation from the speed limit during daytime drive (χ2 = 0.018, p < 0.05).
(Morris, et al., 2015)	USA /2015	Repeated measures within -subjects design, To suggest a better- quality indicator of driving errors around curves in early stage of sleepiness.	20 (9 men, 11 women)	Mean 20.55 (± 2.44) yrs.	One night of total sleep loss	Simulated highway drive on a high fidelity KQ-Vection fixed-base simulator/20-min	5 times of drive from 8 pm. to 10 am.	a) Lateral lane position variability (deviation in lateral lane position), b) SD of Lateral lane position, c) Performance on the curves, e) Heading difference variability; yaw in degrees (the momentary difference between the direction of the vehicle and the tangential direction of the lane).	a) Increase of deviation in lateral lane position (absolute values) F _(4,76)_ = 10.011, p<0.001, η^2^p = 0.345, b) Increase in lateral lane deviation across the night (5 test sessions). c) Increase in vehicle heading differences variability (raw data not absolute values) F_(4,76)_ = 15.989, p<0.001, η2p = 0.345457.
(Jackson, et al., 2016)	Australia /2016	Repeated measures within- subjects design, To compare newer algorithms of driver sleepiness over PERCLOS, to compare impairment of total sleep loss with blood alcohol concentrations	22 (3 men, 19 women)	18–26 yrs., mean 20.8(± 1.9) yrs.	One night of total sleep loss	The AusEd simulated driving task (computer-based divided attention task)/ 30 minutes	Night time	a) SD of lateral position (the distance from middle of left-hand lane), b) Speed variability (variations of speed from 60–80 km/h), c) Mean number of crashes (off road events, stoppage events, truck collisions).	a) Increase in SD of lateral position (from 97.98 in baseline to 115.40 cm in sleep deprived condition ((F = 6.81, *P* = 0.016), b) No change in speed variability (*P* = 0.214), c) No change in mean number of crashes (*P* = 0.348).
**Chronic sleep loss**									
Mild sleep loss (1 <sleep loss <2)									
(Matthews, et al., 2012(b))	Australia /2012	Between-groups design, Comparison of control group with medium and severe sleep deprived groups	41 men	Mean 21.8 (±3.8) yrs.	Chronic sleep loss of 1 h and 3 h.	York Driving Simulator/ 10 min	Rotating sleep/wake in to forced descynchrony time, 8–9 times/day.	a) Lateral position (distance from centre of the car to the left lane marker), b) SD of lateral position	a) Lateral position not reported, b) Increase in SD of lateral position in both moderate and severe sleep restriction.
(Garner, et al., 2015)	USA /2015	Repeated measures within-subjects design, effect of sleep loss on adolescent with various vulnerabilities to sleep loss (based on their attentional decline outside driving setting after sleep loss and parent’s report) in various types of roads (urban/suburban vs rural)	17 adolescents (8 men, 9 women)	16–18 yrs., (mean 17.4(± 0.9) yrs.	5 nights of 6.5 h in bed vs 5-night of 10 h in bed (randomised), two nights washout between them	STISIM M300 simulator/2 counterbalanced drives; a 20-min suburban drive, and a 30-min rural drive	Afternoon at 2pm. or 4 pm.	a) SD of lateral position (distance relative to centre line), b) Mean speed, c) SD of speed, d) Occurrence of crashes: 0 = no crash, 1 = crash event.	a) No effect of sleep condition on variables, b) A three-way interaction of sleep x drive x vulnerability (P = 0.019), effect size η^2^ = 0.33; on rural drive, greater SD of lateral position in sleep loss than sleep extension, in urban drive worsened SD of lateral position only in vulnerable people to sleep loss, c) A three-way interaction of sleep x drive x vulnerability (P = 0.015), effect size η^2^ = 0.36; Vulnerable people had less speed during sleep restriction than sleep extension in rural drive.
Moderate sleep loss (2 <sleep loss <4)									
(Matthews, et al., 2012(a))	Australia /2011	Repeated measures, within-subjects design, Comparison of chronic moderate sleep loss with normal sleep	14 men	mean 21.8 (±3.8) yrs.	Chronic sleep loss, 3 h sleep loss (5 h sleep)	York Driving Simulator/10 min	9 times/day, both day and night time during a 7 forced- desynchronized periods of 23.33 h of wake followed by 4.67 h of time in bed)	a) Lane position (distance from centre of the car to the left lane marker), b) SD of lane position, c) Lane violation, d) Line crossing, e) Crash (when centre of the car leaves the road or car hits the adjacent car), f) Mean and SD of speed, g) Mean deviation from the speed limit, h) Speed variability (SD of deviation from speed limit), i) Speed violation (cumulative time that speed was 5 km/h more than speed limit).	a) No effect of day (sleep debt) on mean lane position, b) Increase in SD of lane position by time of day or prior wake times or days with growing sleep debt, c) Increase in lane violation by time of day or prior wake times or days with growing sleep debt, d) Change in mean speed after sleep loss over different days, e) Increase in SD of speed after sleep loss at different times of day (mostly after nadir) and on different days, but not at various prior wake times, f) Increase in deviation from speed limit and speed violation by the ‘Day’ variable, capturing the growing sleep debt, g) Speed variability not reported explicitly, but increased by time of day (from 180 degrees after nadir to circadian phase 60 degree) per the figure.
(Kosmadopoulos, et al., 2015)	Australia /2015	Repeated measures between-subjects design, to examine the effect of sleep loss on neurobehavioral and subjective tasks	32 men	Mean 22.8(± 2.9) yrs.	9 days of forced-desynchronized protocol with 4h sleep per 24 h	York desktop driving simulator/10 min	5 times in every 24 hours, every 2.5 h, beginning 1.5 h after awakening	SD of lateral position (distance in metres from the centre point of the car to the centre of a two-lane road)	a) SD of lateral position was most sensitive to sleep loss F (1, 30) = 38.52, *P*<0.001, and circadian phase F(5,534) = 61.48, *P*<0.001, and interaction of circadian phase with sleep dose F(5,534) = 31.98, *P*<0.001, b) Large effect sizes of sleep loss (f^2^ = 1.28) and circadian phase (f^2^ = 0.58) on SD of lateral position. c) A large interaction of circadian phase with sleep loss on SD of lateral position (f^2^ = 0.85) during circadian nadir.

^a^Standard deviation

^b^Same as study of Verster., et al 2011

^c^Same as study of Contardi., et al 2004

All papers reported on more than one outcome measure. Many of the papers did not directly report the standard estimates of effect size such as partial eta square or Cohen’s D, Cohen's f^2^, coefficient of correlation (r), or coefficient of determination (r^2^). Instead, they reported unstandardized effect sizes (the differences in outcome variables in the original units of variables), and some papers reported results as confidence intervals. Only four papers [61, 63, 67, 69], reported the effect sizes as Cohen’s d, Cohen's f^2^, or partial eta square. Different outcome measures were reported including lane crossings events, lateral position variables, speed variables, and crash events. As it is obvious from Table 3, there was a great variability in the methodological profiles of the studies presenting challenges for comparison of the effects of sleep loss and the generalisability of findings. More specifically, despite the prior intention of conducting meta-analyses in the protocol, the heterogeneity of the studies and incomplete reporting of effect sizes made this inappropriate.

### Findings of the reviewed papers

#### Lateral position variables

As Table 3 shows lane position (lateral position) had different definitions, referring to the distance from a certain point on the car (i.e. the centre of the car, right side of the right front wheel) to some reference point on the road (i.e. roadway midline, one of lane markers, left lane marker). While, mean lateral position was not the primary outcome in most studies, and reported only in two studies with no effect of sleep loss on this outcome [61, 68], the standard deviation of lateral position was the most frequently reported outcome after both acute and chronic sleep loss (nine papers; [26, 60, 61, 63, 65–69]), representing variability in lane positioning.

While moderate acute sleep loss (3 h) increased the standard deviation of lateral position (large effect size, in a short simulated drive of 30 min [61]), with unclear changes in longer duration of drives [62, 64], severe acute sleep loss of 5 to 5.5 h increased this outcome measure in both short (30 min) [61] and long drives [26], by 1.2 fold after the 30^th^ min of 1.5-h drive [26]. One night of total sleep loss also increased the standard deviation of lateral position in short simulated drives (30 min), reported either as a large effect size [69], or an increase of 17 cm [65] to 20 cm [68, 70]. Similarly, chronic mild (1 to 2 h) [60, 67] or moderate (3 to 4 h) sleep loss [63, 66], both significantly increased the standard deviation of lateral position [60, 63, 66, 67] in short simulated drives of less than 20 min.

Overall, these nine simulator papers reported an adverse effect of sleep loss, except for one study [67] reporting no significant change in this outcome associated with sleep loss, while none of on-road studies, with severe sleep loss (6 h) and longer duration of drives (1.5–2 h) have reported this outcome measure [56–59].

#### Lane crossings

Lane crossings (inappropriate line crossings) was the second most frequently reported variable, appearing in eight papers [56–59, 61, 62, 64, 66], and variously defined as crossing one lateral lane marker, leaving the road by all four wheels, and running off the road at least by two wheels.

In simulated driving paradigms, lane crossings increased significantly under different combinations of sleep loss and duration of drive. Both moderate (3 h) and severe acute sleep losses (5 h) in both short (30 min) [61] and long drives (the last 30 min of a 1.5-h drive) [62], increased number of lane crossings and the cumulative number of lane crossings (6-h sleep loss, 2-h simulated drive)[56]. There was also a positive correlation between lane crossings and distraction (defined as looking away from the main road way for more than 3 s) has also been reported in long simulated drives of 2-h under both moderate (3 h) and severe (5 h) sleep loss [64]. Similarly, a chronic moderate sleep loss (3 h) in a forced desynchrony protocol increased lane crossings in a short simulated drives of 10 min[66].

In on-road studies severe acute sleep loss (6 h) increased the number of line crossings [56], as well as the cumulative number of line crossings per person [57] during 6 and 5 episodes of a 1.5-h drive per day respectively [57], as well as longer drives of 2 h, 4 h and 8 h when compared with the reference driving session (9–10 p.m.) [58, 59]. In general, line crossings were reportedly increased after a variety of sleep loss and drive time combination.

#### Speed variables

A variety of speed variables were reported in six studies [61, 65–68, 70]. Moderate to severe acute sleep loss (3–5 h) [61] or even a total sleep loss [65], in short simulated drives of 30 min, did not impair mean deviation from speed limit [61] nor standard deviation of deviation from speed limit (speed variability) [61, 65]. In two other studies, with the same drive times, total sleep loss did not change mean speed and speed variability, but significantly increased mean deviation from speed limit [68, 70].

Chronic mild sleep loss (1.5 h) over 5 nights in short simulated drives (20-30-min) did not affect mean speed and speed variability [67]. Conversely, chronic (9-d force-desynchrony) moderate sleep loss (3 h) in short 10-min simulated drives, not only resulted in increases in variables such as deviation from the speed limit and speed violation (cumulative time of having a speed 5 km/h more than speed limit) as sleep debt accumulated over 9 days [66], but also an increase in speed variability at night time (effect of circadian phase) [66]. Overall, speed variables were less frequently and inconsistently found to respond to combinations of various types or severities of sleep loss and durations of drives.

#### Crash events

Crash events were reported in four papers [65, 67, 68, 70], either with no explicit definition [67, 68, 70], or defined as driving off the road, stoppage events, or truck collisions [65]. From three studies, while acute total sleep loss in short simulated drives of 30 min did not change number of crashes in one study [65], there were significant increases in two other studies [68, 70]. Chronic mild (1.5 h) sleep loss did not also change the presence of crashes in 20-min simulated drives [67]. These findings suggest an inconsistency in crash events under various sleep deprivation paradigms.

#### Effect of circadian drive for sleepiness on the findings

The circadian-mediated drive for sleep (time-of-day) contributed to impairments of some outcomes during the circadian nadir (typically the early morning hours) or in the afternoon. The time-of-day effect was reported in three forced-desynchronized studies that applied a 1 to 2-h [60], a 3- h [66] or a 4-h [63] sleep deprivation and a 10-min drive time in their protocols. In one study, the effect of prior wake time on standard deviation of lateral position was significantly greater at the circadian phase 60° after nadir (2 h after awakening) when compared with circadian phase 180° after nadir (7 h after awakening) [60]. In another study, standard deviation of lateral position had a significant rise at circadian phase 180° after nadir (7 h after awakening), as opposed to circadian phase 60° after nadir (2 h after awakening) [66]. In a more recent study a large effect of circadian phase was found on standard deviation of lateral position during circadian nadir (circadian phase 0°) [63]. Greater impairments at circadian phase 60° after nadir (2 h after awakening) have also been reported in in the speed variability [66].

The interaction between sleep loss and time-of-day is an important point to consider. Forced desynchronized studies support an effect of sleep restriction on performance, but one that is mediated by circadian phase position.

### Direction of effects

The possibility of statistically combining the quantitative results by conducting a formal meta-analysis was explored. However, due to insufficiency, inconsistency, and non-comparability of the unstandardized reported effects, it was not feasible to combine the data to obtain a single pooled estimated effect size for each outcome. Instead then, this review determined and summarised the direction of effects of sleep loss on each outcome measure, as has been adopted in other sleep-related systematic reviews [71].

**Fig 3:**
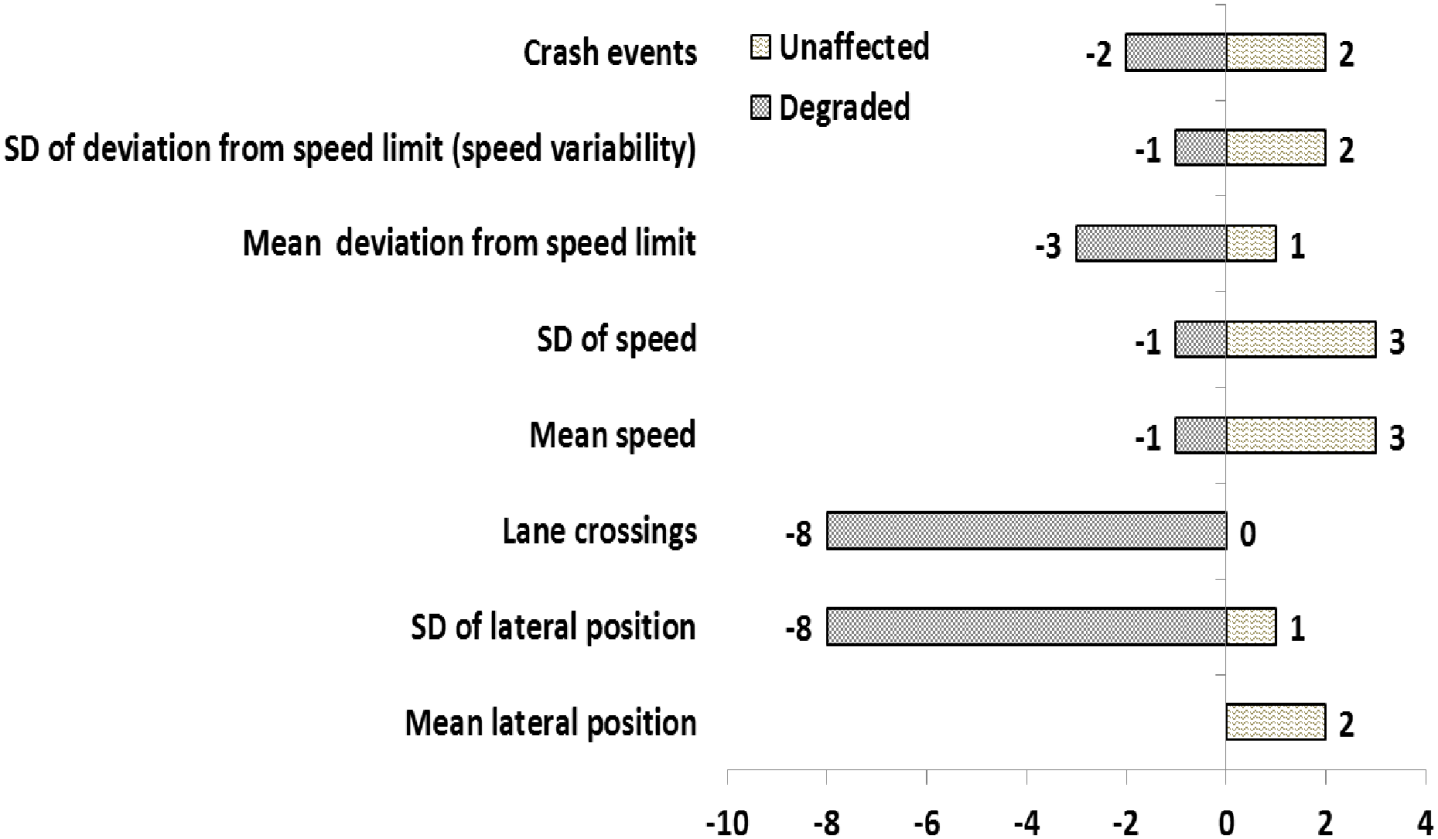
Direction of effects of sleep deprivation on driving performance outcome measures.

Fig 3 shows the number of studies that reported each outcome as either impaired (identified by negative numbers on the left side) or unaffected (identified by positive numbers on the right side). The most commonly reported outcome measures were standard deviation of lateral position and lane crossings, respectively. Mean lateral position was the least frequently reported outcome, since it was not a primary outcome of interest and was only monitored to obtain lane crossing or standard deviation of lateral position. The standard deviation of lateral position and lane crossings were consistently reported to be impaired by sleep loss, while there were inconsistencies in speed related variables and crash events.

### Quality of individual papers and the body of evidence

A summary of methodological elements (strengths and weaknesses) of the reviewed studies, that were considered for developing the GRADE criteria, is presented in the supplementary information (S1 Table). The GRADE criteria for rating the quality of each outcome measure in the individual papers are represented in Table 4.

**Table 4:** The GRADE criteria for rating the quality of body of evidence for each outcome measure.

Design quality	Design type and quality score	Factors decreasing the quality			Score deducted	Factors increasing the quality	Score added
**High**	RTC; score 4	Risk of bias	Inappropriate eligibility criteria	Inclusion people with: Shift-work., Professional driving, Travel to different time zone in the last three months, Sleep disorders, Smoking, Habitual heavy caffeine consumption, Caffeine avoidance, Alcohol abuse (more than two standard drinks per day), Inclusion people from a specific place only (e.g. university students only)	-1	Control for exposure and inclusion criteria: Strong control of sleep loss before test Strong inclusion criteria	+1
**Low**	Observational study: Experimental or longitudinal; score 3		Inadequate control for confounders	Age, Gender, Driving experience, Inter-individual differences in sensitivity to sleep loss Presence of circadian drive or time-on-task effect for sleepiness		Confounders: Residual confounders that are decreasing the estimated effect size (listed in the quality- decreasing factors), Strong control for confounders	+1
	Observational study: Quasi-experimental or cross-sectional study; score 2		Reporting bias	Unreported results for the outcome measure		Certainty: Large effect size, Large sample size, Objectively confirming sleepiness (EEG), Control for distraction	+1
			Conflict of interest	Study being funded by an organisation or industry increasing risk of reporting bias			
	Other designs; score 0		Flaws in measuring sleepiness and outcome	Inadequate monitoring sleep-wake before test, Inadequate control for stimulants before (sleep-wake monitoring time) and during test, Inadequate monitoring sleepiness during test (no wake EEG) Practice effect Unclear definition of outcome Inappropriate measurement of the outcome (including poor control for distraction)			
		Imprecision (uncertainty)	Small sample size affecting generalisability		-1		

The quality of each outcome measure within individual papers and across papers (body of evidence) is rated against the GRADE criteria in S2 Table. Clearly, each individual paper has been assigned different quality scores for different outcomes.

Table 5 represents the ranking of the body of evidence for the quality of each outcome. Of the body of evidence that infrequently reported driving performance outcomes such as mean lateral position, deviation from speed limit, speed variability and crash events, all ranked very low quality suggesting a very low level of confidence of proximity of estimated effect of sleep deprivation on these outcomes to real effect. The body of evidence that frequently reported other outcomes such as standard deviation of lateral position, lane crossing, mean speed and standard deviation of speed were ranked low-quality evidence with a limited confidence of validity of estimated effect. None of the reported outcomes came from a medium or high-quality body of evidence.

**Table 5:** Ranking of the quality of the body of evidence for each driving performance outcome measure.

	Quality of body of evidence			
Outcome measure	Very low 0<OGS<1	Low 1<OGS<2	Medium 2<OGS<3	High 3<OGS
Mean lateral position	*			
SD ^a^ of lateral position		*		
Lane crossing		*		
Mean speed		*		
SD of speed		*		
Deviation from speed limit	*			
Speed variability	*			
Crash events	*			

^a^ Standard deviation

## Discussion

Based on the PRISMA-based systematic search in this review there is only limited (16 peer-reviewed original papers) available evidence, with no systematic reviews, for impact of sleep loss on driving performance of young drivers over the last decade. This limited literature suffers from considerable inconsistencies in study designs, sample sizes, sleep deprivation regimes, definition and measurement of outcomes, driving settings, time-of-day, duration of drives, control for confounding factors, reporting of methodologies and results and magnitudes of effects. This heterogeneity of multiple study aspects and reported outcomes limits the generalisability of the findings and ability to conduct a meta-analysis.

Lack of high-quality evidence in the existing literature, when applying the GRADE approach for quality ranking, could be mainly due to weak design, risk of bias and imprecision. The study designs included some robust quasi-experimental cross-over, within-groups, or between-groups repeated measures designs, but no randomized control trials (RCTs), nor large-scale studies or strong experimental designs. While “risk of bias” stemmed from inadequate monitoring of sleepiness while conducting the experiment and presence of task practice effect, “imprecision” (uncertainty) arose from small sample sizes with only male participants, possibly due to the over-representation of men in road crashes, or because of attempts to control for sex differences in response to sleep loss.

The standard deviation of lateral position and lane crossings were the two most commonly examined and predominantly impaired outcomes in this review. The findings suggest that the standard deviation of lane position is sensitive to prior wake period, time of day, and the day of sleep deprivation [66], with significant impairments of under acute [61] and chronic sleep loss [60, 67]. Similarly, lane crossings was reported to increase after acute sleep loss [56]. These findings are in agreement with previous reports that lateral lane position and steering wheel variables are the most sensitive outcomes to sleep loss, both of which could result in lane crossings or hitting adjacent cars [55]. However, none of the reviewed papers reported findings for steering wheel variables, sufficient to enable any determination here on the utility of those variables. These findings therefore have limited reliability and suffer from a low quality of body of evidence suggesting a limited level of confidence in these two outcomes.

Speed related outcomes and crash events in this review both responded to sleep loss inconsistently. For instance, mean and standard deviation of speed as well as deviation from speed limit did not change after acute sleep loss, but significantly deteriorated after chronic sleep loss. Likewise, crash events in some studies did not change after acute sleep loss, but in other studies increased both in acute and chronic sleep loss. These findings on the one hand do not suggest a clear direction for effect of sleep loss, and on the other hand were graded as low quality and a carry a very limited confidence in their accuracy (reliability).

In summary, a small body of evidence is currently supporting the consequences of sleep loss on young drivers’ performance, with considerable variety in the study designs, outcome measures, severity of sleep loss and methodologies. The reviewed studies do not suggest a robust and generalized conclusion for the type and magnitude of the effects. Consistent increases in standard deviation of lateral position and line crossing events were identified, but this was not the case for crash events and other speed-related outcomes. There is also no clear distinction between impact of sleep loss and circadian misalignment, since the confounding effects of circadian contributors to sleepiness have not been considered in the majority of these studies. Even these limited findings are questionable as the evidence is from very low to low quality studies as assessed by the GRADE criteria.

To draw a unified conclusion on the effect of sleep loss on young driver’s performance, it is crucial for future studies to initially adopt higher quality experimental designs, including the RCTs to test interventions or superior epidemiological methods to ensure adequate power. Next, common protocols and consistent metrics should be taken in consideration when developing methodologies. Young female drivers should be included in studies intended to represent the driving population and to further research into any gender differences in response to sleep loss. The ecological limitations of driving simulators on the one hand, and progressive developments in driver and in-vehicle monitoring technologies on the other hand, suggest a need to shift from simulators towards on-road measurements. Lastly, best practice reporting protocols as outlined in the GRADE guidelines and the PRISMA Statement should be considered when reporting the findings to enable meta-analyses.

## Supporting information

S1 Table Methodological elements of papers considered for quality rating. (DOCX)

S2 Table Quality of individual papers and body of evidence based on the GRADE criteria. A single GRADE score for a given outcome within an individual paper and an Overall Grade Score (OGS) for the body of evidence for that outcome are presented in the last two columns of this table. The upgrading and downgrading elements have been highlighted in yellow. The outcomes that have been reported once could not be assigned any OGS and are marked as NA in the table.
(DOCX)

S1 PRISMA Checklist PRISMA checklist. (DOCX)
