# Supplementary material for: The effects of sleep loss on young drivers’ performance: A systematic review
Source: PLoS One. 2017 Aug 31;12(8):e0184002. doi: 10.1371/journal.pone.0184002 (PMC5578645; doi:10.1371/journal.pone.0184002)
Supplement: S1 Table — (DOCX) [file pone.0184002.s001.docx]

**S1 Table. Methodological Elements of Papers Considered for Quality Rating**

| **Paper** | **Methodological strengths and flaws** | **Factors considered for quality rating** |
| --- | --- | --- |
| (Philip, et al., 2005_(b)_) | 1. The reference point for measuring lateral position (centre or lateral side of the car etc.) was not reported, 2. No measurement for possible distraction sources (confounders), 3. No wake EEG measurement for confirming sleepiness, 4. Not known if participants were professional drivers, shift workers, or had experienced recent time-zone travel, 5. Consumption of caffeine, alcohol, and other stimulants/sedatives was not reported, 6. Only males were included without any clear rationale for that, 7. No criterion for quantifying driver experience (just yearly driving distance is given), 8. Small sample size | 1. No wake EEG measurement for confirming sleepiness, 2. Small sample size |
| (Philip, et al., 2005_(a)_) | 1. The reference point for measuring lateral position (centre or lateral side of the car etc.) was not reported, 2. No measurement for possible distraction sources (confounder), 3. No wake EEG measurement for confirming sleepiness, 4. Not known if participants were professional drivers, shift workers, or had experienced recent time-zone travel, 5. Consumption of caffeine, alcohol, and other stimulants/sedatives was not reported, 6. Only males were included without any clear rationale for that, 7. No criterion for quantifying driver experience (just yearly driving distance is given), 8. Small sample size | 1. No wake EEG measurement for confirming sleepiness, 2. Small sample size, |
| (Matthews, et al., 2012_(b)_) | 1. Only males were included without any clear rationale for that, 2. No measurement for possible distraction sources (confounder), 3. No wake EEG measurement for confirming sleepiness, 4. Consumption of caffeine, alcohol, and other stimulants/sedatives was not reported, 5. No criterion for quantifying driver experience (just yearly driving distance is given), 6. Large sample size, 7. Presence of learning effect for lane deviation in the control condition | 1. No wake EEG measurement for confirming sleepiness, 2. Big sample size, 3. Presence of learning effect for lane deviation in the control condition, |
| (Matthews, et al., 2012_(a)_) | 1. Only males were included without any clear rationale for that, 2. No measurement for possible distraction sources (confounder), 3. No wake EEG measurement for confirming sleepiness, 4. No criterion for quantifying driver experience (just yearly driving distance is given), 5. Consumption of caffeine, alcohol, and other stimulants/sedatives was not reported, 6. Small sample size, | 1. No wake EEG measurement for confirming sleepiness, 2. Small sample size |
| (Pizza, et al., 2004) | 1. The study design is not reported (within-participant design), 2. The reference point (centre or lateral side of the car etc.,) has not been reported, 3. Age range is not specified and method of control for age is unknown, 4. No criterion for quantifying driver experience (just yearly driving distance is given), 5. No measurement for possible distraction sources (confounder), 6. No wake EEG measurement for confirming sleepiness, 7. Small sample size | - 1. No wake EEG measurement for confirming sleepiness,  1. Small sample size |
| (Lowden, et al., 2009) | 1. Small sample size, 2. Good control for confounders, 3. Wake EEG, EOG measurement for confirming sleepiness | 1. Small sample size, 2. Good control for confounders, 3. Wake EEG measurement for confirming sleepiness, |
| (Rupp, et al., 2004) | 1. The study design is not reported (between-participant design), 2. No criterion for quantifying driver experience (just yearly driving distance is given), 3. Good control for sleep deprivation, 4. No wake EEG measurement for confirming sleepiness, 5. No measurement for possible distraction sources (confounder) | 1. Good control for sleep deprivation, 2. No wake EEG measurement for confirming sleepiness, |
| (Sagaspe, et al., 2008) | 1. Type of lane crossing, partial (one wheel) or total (two wheel), was not reported, 2. Good control for sleep deprivation before and during study, 3. No wake EEG measurement for confirming sleepiness, 4. No measurement for possible distraction sources (confounder), 5. Results of the simulator might not be generalizable to real-life driving, except perhaps on a group level, 6. Not known if participants were professional drivers, shift workers, or had experienced recent time-zone travel, 7. Consumption of caffeine, alcohol, and other stimulants/sedatives was not reported, 8. Only males were included without any clear rationale for that, 9. Small sample size | 1. Good control for sleep deprivation before and during study, 2. Small sample size |
| (Anderson & Horne, 2013) | 1. The side of the road to drive was not mentioned, 2. The study design was not mentioned properly, 3. Control for driver experience, 4. Small sample size, 5. Good control for distraction, 6. Good control for sleep deprivation, 7. No wake EEG for confirming sleepiness, | 1. Small sample size, 2. Good control for distraction, 3. Good control for sleep deprivation, 4. No wake EEG for confirming sleepiness, |
| (Filtness, et al., 2012) | 1. Good sample size, 2. Control for driving experience, 3. The rationale for choosing only males was mentioned, 4. Good control of driver sleep deprivation before and during test, 5. Control for distraction by filming driver face, 6. Wake EEG and EOG | 1. Good sample size, 2. Good control of driver sleep deprivation before and during test, 3. Control for distraction by filming driver face, 4. Wake EEG and EOG, |
| (Jackson, et al., 2016) | 1. The reference point for measuring lateral position (centre or lateral side of the car etc.) was not reported, 2. No wake EEG for confirming sleepiness, 3. Small sample size, 4. No control of distraction when driving 5. The effect of circadian drive (time of day) for sleepiness is not distinguished from homeostatic drive (sleep loss) | 1. No wake EEG for confirming sleepiness, 2. Small sample size, |
| (Kosmadopoulos, et al., 2015) | 1. Small sample size (only 16 for sleep deprived group) 2. Good control for sleep deprivation (Actigraphy) 3. Only males were included without any clear rationale for that 4. Good control of sleep-wake during 2 weeks forced desynchrony using closed circuit cameras 5. No wake EEG during drives 6. No control of distraction when driving | 1. Small sample size 2. Good control for sleep deprivation (Actigraphy) 3. No wake EEG for confirming sleepiness, |
| (Garner, et al., 2015) | 1. The reference point for measuring lateral position (centre or lateral side of the car etc.) was not reported, 2. No alcohol and caffeine intake, shift work or travel as inclusion criteria 3. Good monitoring sleep-wake with Actigraphy 4. No wake EEG measurement for confirming sleepiness, 5. No control of distraction when driving | 1. No wake EEG measurement for confirming sleepiness, 2. Good control for sleep deprivation (Actigraphy) |
| (Morris, et al., 2015) | - 1. No inclusion criterion for habitual caffeine use   2. No monitoring wake EEG during drives   3. Good monitoring alcohol and caffeine usage during test night   4. Poor control for sleep and wake before test ( no Actigraphy)   5. No control of distraction when driving | 1. No wake EEG measurement for confirming sleepiness |
