# Supplementary material for: The effects of sleep loss on young drivers’ performance: A systematic review
Source: PLoS One. 2017 Aug 31;12(8):e0184002. doi: 10.1371/journal.pone.0184002 (PMC5578645; doi:10.1371/journal.pone.0184002)
Supplement: S2 Table — A single GRADE score for a given outcome within an individual paper and an Overall Grade Score (OGS) for the body of evidence for that outcome are presented in the last two columns of this table. The upgrading and downgrading elements have been highlighted in yellow. The outcomes that have been reported once could not be assigned any OGS and are marked as NA in the table. (DOCX) [file pone.0184002.s002.docx]

S2 Table^a^. Quality of Individual Papers and Body of Evidence Based on the GRADE Criteria

| Outcome | Authors/  Code | Downgrading factors for study design, risk of bias (limitations in execution) and imprecision | | | | | | | | | | | | | | Upgrading factors | | | | | | | | Overall GRADE  Score | |
| --- | --- | --- | --- | --- | --- | --- | --- | --- | --- | --- | --- | --- | --- | --- | --- | --- | --- | --- | --- | --- | --- | --- | --- | --- | --- |
|  |  | **Study design** | | | **Risk of bias for other limitations** | | | | **Risk of bias for inappropriate measurement of sleepiness and outcome** | | | | | **Imprecision** | | **Exposure and inclusion criteria (+1)** | | **Confounder**  **(+1)** | | **Certainty (+1)** | | | | **Overall GRADE score (paper)** | **Overall GRADE Score (body of evidence)** |
|  |  | RTC (+4) | Longitudinal (+3) | Quasi- experiment (+2) | Inappropriate /unclear eligibility criteria | Inappropriate /unclear control for confounders | Reporting bias | Conflict of interest | Inadequate monitoring sleep-wake before test | Inappropriate /unclear control for stimulants before and during test | Inadequate monitoring sleepiness during test (wake EEG) | Practice effect | Unclear definition/ poor measurement of outcome | Ungeneralizable findings | Small sample size | Strong control of sleep loss before test | Strong inclusion criteria | residual confounding decreasing the estimated effect | Strong control for confounders | Large effect size | Large sample size | Objective measurement of sleepiness (EEG) | Control for distraction |  |  |
| **lateral position** | (Philip, et al., 2005_(b)_) |  |  | * | * | * | * |  |  | * | * |  | * |  | * |  |  | * |  |  |  |  |  | 0 | 0.54 |
|  | (Philip, et al., 2005_(a)_) |  |  | * | * | * | * | * |  |  | * |  | * |  | * |  |  | * |  |  |  |  |  | 0 |  |
|  | (Matthews, et al., 2012_(b)_) |  |  | * |  | * | * | * |  |  | * |  |  |  |  |  |  | * |  |  | * |  |  | 2 |  |
|  | (Matthews, et al., 2012_(a)_) |  |  | * |  | * |  | * |  |  | * |  |  |  | * |  |  | * |  |  |  |  |  | 0 |  |
|  | (Pizza, et al., 2004) |  |  | * |  | * | * |  |  |  | * |  | * |  | * |  |  | * |  |  |  |  |  | 0 |  |
|  | (Lowden, et al., 2009) |  |  | * |  | * | * | * |  |  |  |  |  |  | * |  |  |  | * |  |  | * |  | 2 |  |
|  | (Jackson, et al., 2016) |  |  | * |  |  |  |  |  |  | * |  | * |  | * |  |  |  |  |  |  |  |  | 0 |  |
| **Mean lateral deviation from centre of the road** | (Philip, et al., 2005_(b)_) |  |  | * | * | * | * |  |  |  | * |  | * |  | * |  |  | * |  |  |  |  |  | 0 | 0.74 |
|  | (Pizza, et al., 2004) |  |  | * |  | * |  |  |  |  | * |  | * |  | * |  |  | * |  |  |  |  |  | 0 |  |
|  | (Rupp, et al., 2004) |  |  | * |  | * |  |  |  |  | * |  |  |  |  | * |  | * |  |  |  |  |  | 2 |  |
| **SD of lateral position** | (Philip, et al., 2005_(b)_) |  |  | * | * | * | * |  |  |  | * |  | * |  | * |  |  | * |  |  |  |  |  | 0 | 1.31 |
|  | (Pizza, et al., 2004) |  |  | * |  | * |  |  |  |  | * |  | * |  | * |  |  | * |  |  |  |  |  | 0 |  |
|  | (Matthews, et al., 2012_(b)_) |  |  | * |  | * |  | * |  |  | * | * |  |  |  |  |  | * |  |  | * |  |  | 1 |  |
|  | (Lowden, et al., 2009) |  |  | * |  |  |  | * |  |  |  |  |  |  | * |  |  |  | * |  |  | * |  | 2 |  |
|  | (Matthews, et al., 2012_(a)_) |  |  | * |  | * |  | * |  |  | * |  |  |  | * |  |  | * |  |  |  |  |  | 0 |  |
|  | (Rupp, et al., 2004) |  |  | * |  | * |  |  |  |  | * |  |  |  |  | * |  | * |  | * |  |  |  | 3 |  |
|  | (Jackson, et al., 2016) |  |  | * |  |  |  |  |  |  | * |  | * |  | * |  |  |  |  |  |  |  |  | 0 |  |
|  | (Kosmadopoulos, et al., 2015) |  |  | * |  | * |  |  |  |  | * |  | * |  | * | * |  | * |  | * |  |  |  | 2 |  |
|  | (Garner, et al., 2015) |  |  | * | * |  |  |  |  |  | * |  | * |  |  | * |  |  |  | * |  |  |  | 3 |  |
|  | (Morris, et al., 2015) |  |  | * | * |  |  |  | * |  | * |  | * |  |  |  |  |  |  | * |  |  |  | 2 |  |
| **Lane crossing** | (Sagaspe, et al., 2008) |  |  | * | * | * |  | * |  | * | * |  | * | * | * | * |  | * |  |  |  |  |  | 0 | 1.40 |
|  | (Anderson & Horne, 2013) |  |  | * |  |  |  |  |  |  | * |  | * |  | * | * |  |  |  |  | * |  | * | 3 |  |
|  | (Matthews, et al., 2012_(a)_) |  |  | * |  | * |  | * |  | * | * |  |  |  | * |  |  | * |  |  |  |  |  | 0 |  |
|  | (Matthews, et al., 2012_(b)_) |  |  | * |  | * |  | * |  |  | * | * |  |  |  |  |  |  |  |  | * |  |  | 1 |  |
|  | (Philip, et al., 2005_(b)_) |  |  | * | * | * |  |  |  |  | * |  | * |  | * |  |  | * |  |  |  |  |  | 0 |  |
|  | (Philip, et al., 2005_(a)_) |  |  | * | * | * |  | * |  |  | * |  | * | * | * |  |  | * |  |  |  |  |  | -1 |  |
|  | (Lowden, et al., 2009) |  |  | * |  |  | * | * |  |  |  |  |  |  | * |  |  |  | * |  |  | * |  | 2 |  |
|  | (Rupp, et al., 2004) |  |  | * |  | * |  |  |  |  | * |  |  |  |  | * |  | * |  | * |  |  |  | 3 |  |
|  | (Filtness, et al., 2012) |  |  | * |  |  |  |  |  |  |  |  |  |  |  | * |  |  |  |  | * | * | * | 6 |  |
|  | (Pizza, et al., 2004) |  |  | * |  | * |  |  |  |  | * |  | * |  | * |  |  | * |  |  |  |  |  | 0 |  |
| **Mean and *SD of speed** | (Pizza, et al., 2004) |  |  | * |  | * |  |  |  |  | * |  | * |  | * |  |  | * |  |  |  |  |  | 0 | 1.91 |
|  | (Lowden, et al., 2009) |  |  | * |  |  | * | * |  |  |  |  |  |  | * |  |  |  | * |  |  | * |  | 2 |  |
|  | (Garner, et al., 2015) |  |  | * | * |  |  |  |  |  | * |  |  |  |  | * |  |  |  | * |  |  |  | 3 |  |
| **Deviation from speed limit** | (Pizza, et al., 2004) |  |  | * |  | * |  |  |  |  | * |  | * |  | * |  |  | * |  |  |  |  |  | 0 | 0.70 |
|  | (Matthews, et al., 2012_(a)_) |  |  | * |  | * |  | * |  |  | * |  |  |  | * |  |  | * |  |  |  |  |  | 0 |  |
|  | (Rupp, et al., 2004) |  |  | * |  | * |  |  |  |  | * |  |  |  |  | * |  | * |  |  |  |  |  | 2 |  |
| **Speed variability** | (Rupp, et al., 2004) |  |  | * |  | * |  |  |  |  | * |  |  |  |  | * |  | * |  |  |  |  |  | 2 | 0.52 |
|  | (Matthews, et al., 2012_(a)_) |  |  | * |  | * |  | * |  |  | * |  |  |  | * |  |  | * |  |  |  |  |  | 0 |  |
|  | (Jackson, et al., 2016) |  |  | * |  |  |  |  |  |  | * |  | * |  | * |  |  |  |  |  |  |  |  | 0 |  |
| **Number of crashes** | (Jackson, et al., 2016) |  |  | * |  |  |  |  |  |  | * |  | * |  | * |  |  |  |  |  |  |  |  | 0 | 0 |
|  | (Pizza, et al., 2004) |  |  | * |  | * |  |  |  |  | * |  | * |  | * |  |  | * |  |  |  |  |  | 0 |  |
| **Speed violation** | (Matthews, et al., 2012_(a)_) |  |  | * |  | * |  | * |  |  | * |  |  |  | * |  |  | * |  |  |  |  |  | 0 | NA |
| **Distraction** | (Anderson & Horne, 2013) |  |  | * |  |  |  |  |  |  | * |  |  |  | * | * |  |  |  |  | * |  | * | 3 | NA |
| **Steering wheel angle** | (Lowden, et al., 2009) |  |  | * |  |  | * | * |  |  |  |  |  |  | * |  |  |  | * |  |  | * |  | 2 | NA |
| **Heading difference variability** | (Morris, et al., 2015) |  |  | * | * |  |  |  | * |  | * |  | * |  |  |  |  |  |  | * |  |  |  | 2 | NA |

^a^ The factors considered for downgrading and upgrading the GRADE scores are presented in a shaded form.
